# A Relativistic Adaptive Gradient Descent Enhanced SPGD Algorithm for Wavefront Sensorless Adaptive Optics

**DOI:** 10.3390/mi17080958

**Published:** 2026-08-13

**Authors:** Huizhen Yang, Lingzhe Tang, Peng Chen, Chen Sun, Xinyu Xiao, Zhiguang Zhang, Jiacheng Zhou

**Affiliations:** 1 National Laboratory on Adaptive Optics, Chengdu 610209, China; yanghz@jit.edu.cn; 2 Engineering School of Network and Telecommunications, Jinling Institute of Technology, Nanjing 211169, China; 2307010003@stu.jit.edu.cn (L.T.); sun15252505775@163.com (C.S.); 17862260728@163.com (X.X.); 13506233159@163.com (J.Z.); 3 School of Electronic and Engineering, Jiangsu Ocean University, Lianyungang 222005, China; zhangzhiguang10@mails.ucas.ac.cn

**Keywords:** adaptive optics system, deformable mirror, stochastic parallel gradient descent, RAD-SPGD, wavefront correction

## Abstract

Deformable mirrors (DMs) serve as the core wavefront correction devices in wavefront sensorless adaptive optics (AO) systems, and their performance is predominantly determined by the convergence speed and stability of the control algorithm. Although the stochastic parallel gradient descent (SPGD) algorithm is extensively used for wavefront sensorless AO control, its slow convergence limits real-time wavefront correction. To address this issue, the RAD-SPGD algorithm is put forward by integrating the relativistic adaptive gradient descent (RAD) optimizer into the conventional SPGD algorithm. A wavefront sensorless AO system with a 97-element MEMS deformable mirror was established to evaluate the proposed algorithm under different turbulence levels, and physical experiments were carried out for verification. The convergence performance is evaluated by the number of iterations needed for the Strehl ratio (SR) to reach 80% of its maximum value. Simulation results demonstrate that the proposed algorithm improves the correction speed by approximately 56% on average compared with the conventional SPGD algorithm, while experimental results show an improvement of approximately 28.6%. Moreover, dynamic turbulence experiments demonstrate enhanced turbulence adaptability and correction stability. These results suggest that the proposed algorithm effectively enhances the closed-loop control efficiency of the 97-element MEMS deformable mirror, offering an effective solution for real-time wavefront sensorless adaptive optics systems.

## 1. Introduction

Adaptive optics (AO) [1,2] is an optical technique that improves imaging quality by compensating for wavefront aberrations caused by atmospheric turbulence, optical imperfections, and other environmental disturbances. Owing to its excellent wavefront correction capability, AO has been widely applied in astronomical observation, biological microscopy, laser beam transmission, free-space optical communication, and high-resolution imaging [3,4,5,6]. Recent advances in adaptive optics have further extended its applications beyond conventional wavefront correction toward more flexible optical field regulation and performance optimization. For example, adaptive optics has been applied in advanced optical manufacturing processes, where accurate wavefront manipulation is essential for improving processing efficiency and precision [7]. Moreover, intensity-based adaptive optics has demonstrated the potential of using intensity information as feedback for optical optimization, providing new possibilities for sensor-independent adaptive correction strategies [8]. The fundamental principle of an AO system is to compensate for aberrated wavefronts using a wavefront corrector. Among the commonly used wavefront correction devices, deformable mirrors (DMs) [9,10,11,12] have become one of the most widely adopted components. In particular, micro-electro-mechanical system (MEMS) deformable mirrors feature a compact structure, high actuator integration, fast response, and high control accuracy. By applying appropriate control signals to individual actuators, the mirror surface can be dynamically deformed to compensate for wavefront aberrations induced by atmospheric turbulence, optical system errors, and other disturbances. For multi-actuator MEMS deformable mirrors, the wavefront fitting capability and closed-loop correction performance are jointly determined by the actuator configuration, response characteristics, and the control algorithm.

Unlike conventional AO systems, wavefront sensorless AO systems eliminate the need for a wavefront sensor and directly construct a closed-loop feedback based on image quality metrics. The control algorithm generates the driving signals for the deformable mirror to compensate for wavefront aberrations. Among the existing control methods, the stochastic parallel gradient descent (SPGD) algorithm [13,14,15] is widely adopted because of its simple implementation, parallel architecture, and strong robustness. However, the conventional SPGD algorithm employs a fixed gain coefficient, which often results in slow convergence, limited adaptability, and local oscillations under varying turbulence conditions or complex wavefront aberrations. These limitations restrict its application in real-time wavefront correction.

Conventional SPGD algorithms usually employ fixed gain coefficients, which may result in slow convergence or insufficient adaptability under complex wavefront aberration conditions. Therefore, an optimization strategy with adaptive parameter adjustment capability is desirable for improving the convergence performance of SPGD-based AO control systems. Relativistic Adaptive Gradient Descent (RAD), proposed by Lyu et al. [16] in 2024, is an adaptive optimization algorithm with high computational efficiency and adaptive parameter adjustment capability. In addition to adaptively adjusting the gain coefficient, RAD introduces a parameter update velocity constraint to alleviate the influence of abnormal gradients, thereby improving optimization stability and promoting faster convergence. These characteristics make RAD a promising optimization strategy for enhancing the convergence performance of SPGD-based control algorithms.

Motivated by the advantages of RAD, this study incorporates the RAD optimizer into the conventional SPGD framework and proposes a RAD-SPGD control algorithm for wavefront sensorless AO systems. A simulation model and a physical experimental platform based on a 97-element MEMS deformable mirror are established to evaluate the proposed algorithm. The convergence speed, correction performance, and adaptability under different turbulence conditions are systematically investigated and compared with those of the conventional SPGD algorithm. The proposed method is expected to improve the real-time closed-loop control performance of wavefront sensorless AO systems.

## 2. RAD-SPGD Control Algorithm

The stochastic parallel gradient descent (SPGD) algorithm is a model-free optimization method that estimates the gradient direction of the control parameters by applying random perturbations and evaluating the corresponding variation in the system performance metric. In a wavefront sensorless adaptive optics system, the control parameter is the voltage vector applied to the deformable mirror, and the optimization objective is to improve the image-quality metric obtained from the imaging detector.

In the k-th iteration, the control voltage vector applied to the *N* actuators of the deformable mirror can be expressed as

(1) $$u_{k}=u_{k-1}+\alpha \nabla u_{k}\nabla J_{k}$$

where $u_{k}={\left\{u_{1},u_{2},\ldots ,u_{N}\right\}}_{k}$ is the control voltage vector in the k-th iteration, alpha is the gain coefficient, $\nabla u_{k}={\left\{\nabla u_{1},\nabla u_{2},\ldots ,\nabla u_{N}\right\}}_{k}$ denotes the random perturbation voltage vector applied to the deformable mirror, and $\nabla J_{k}$ is the variation in the system performance metric.

During each SPGD iteration, a random perturbation voltage $\nabla u_{k}$ is first generated. The positive perturbation voltage $u_{k-1}+\nabla u_{k}$ is applied to the deformable mirror, and the corresponding detector image is acquired to calculate the performance metric $J_{k}^{+}$. Subsequently, the negative perturbation voltage $u_{k-1}-\nabla u_{k}$ is applied, and the corresponding performance metric $J_{k}^{-}$ is obtained. The variation in the performance metric is then calculated as

(2) $$\nabla J_{k}=J_{k}^{+}-J_{k}^{-}$$

The control voltage vector $u_{k}$ is then calculated according to Equation (1) and applied to the deformable mirror. The detector image is acquired to evaluate the correction effect, thereby completing the k-th iteration. If the predefined termination condition is satisfied, the closed-loop control process is terminated, and the correction is completed; otherwise, the system proceeds to the next iteration.

Although SPGD is simple to implement and has strong robustness, its gain coefficient is usually fixed. When the turbulence intensity changes or the wavefront aberration becomes large, a fixed gain cannot simultaneously ensure fast convergence and stable correction, which may lead to slow convergence or local oscillation. To improve the convergence efficiency and correction stability of wavefront sensorless adaptive optics systems, this study introduces the relativistic adaptive gradient descent (RAD) optimizer into the SPGD control framework and proposes the RAD-SPGD control algorithm.

The RAD-SPGD method updates the control parameters by using the long-term stability of a conformal symplectic integrator. In addition, this method combines the exponential moving average with bias correction, making it applicable to general non-convex stochastic optimization problems. This strategy can reduce the influence of abnormal gradient perturbations and stabilize the closed-loop convergence process of the adaptive optics system. The gradient estimate of the first iteration is first obtained according to Equation (2), and the subsequent RAD-SPGD iteration starts from k=2. The RAD-SPGD-based control method for the adaptive optics system mainly consists of the following three stages.

1. First-order momentum update. The first-order momentum represents the exponential moving average of the gradient estimate and reflects the accumulated update direction of the control parameters. In the RAD optimizer, the update velocity of each parameter is adjusted according to the corresponding momentum term. This feature provides parameter-wise adaptive capability and is beneficial for stable convergence in non-convex stochastic optimization. The first-order momentum update rule is given by

(3) $$v_{k}=\beta_{1}v_{k-1}+(1-\beta_{1})\nabla J_{k}$$

where $v_{k}$ denotes the first-order momentum in the k-th iteration, and $\beta_{1}$ is the first-order momentum coefficient.

2. Second-order momentum update. The second-order momentum describes the exponential moving average of the squared gradient estimate and is used to adaptively adjust the update amplitude of the control parameters according to the gradient magnitude. The second-order momentum is updated as

(4) $$y_{k}=\beta_{2}y_{k-1}+(1-\beta_{2}){\left(\nabla J_{k}\right)}^{2}$$

where $y_{k}$ denotes the second-order momentum in the k-th iteration, and $\beta_{2}$ is the second-order momentum coefficient.

During parameter initialization, the first-order momentum v and the second-order momentum y are generally initialized to zero. This zero initialization may introduce estimation bias in the early stage of the algorithm, thereby affecting the accuracy of parameter updates and the stability of system convergence. Therefore, bias correction is required for the momentum terms. Specifically, the first-order and second-order momentum terms are corrected as follows:

$${\hat{v}}_{k}=\frac{v_{k}}{1-\beta_{1}^{k}}$$

(5) $${\hat{y}}_{k}=\frac{y_{k}}{1-\beta_{2}^{k}}$$

where ${\hat{v}}_{k}$ and ${\hat{y}}_{k}$ denote the bias-corrected first-order momentum and second-order momentum, respectively. Through the above correction, the estimation bias introduced by zero initialization in the initial stage of the algorithm can be reduced, thereby improving the stability of the parameter update process.

3. Control parameter update. After the first-order momentum, second-order momentum, and their bias-corrected forms are obtained, the control parameters of RAD-SPGD are further updated. The update rule for the control parameter $u_{k}$ is expressed as

(6) $$u_{k}=u_{k-1}-\frac{\sqrt{1-\beta_{2}^{k}}}{\sqrt{\delta^{2}y_{k}+\zeta_{k-1}}}\alpha \cdot \frac{v_{k}}{1-\beta_{1}^{k}}$$

where $\frac{\sqrt{1-\beta_{2}^{k}}}{\sqrt{\delta^{2}y_{k}+\zeta_{k-1}}}$ denotes the adaptive gain term, $\frac{v_{k}}{1-\beta_{1}^{k}}$ denotes the bias-corrected first-order momentum term, δ denotes the velocity coefficient, which controls the magnitude of the adaptive update by regulating the gradient normalization term. A larger enhances the gradient normalization effect, reduces the update amplitude in each iteration, and improves the stability of the algorithm. In contrast, a smaller weakens the update constraint and increases the response speed, but may introduce a higher risk of oscillation during the iterative process. Therefore, plays a balancing role between convergence speed and stability in RAD-SPGD. $\zeta_{k-1}$ represents a stability factor introduced into the adaptive gradient normalization term. It regulates the denominator of the update equation to prevent numerical instability caused by excessively small values and limits abnormal update steps. In both the numerical simulations and physical experiments, the parameters were set as $\beta_{1}$ = 0.9, $\beta_{2}$ = 0.999, α = 0.04 and δ = 0.75. The four parameters were initially selected according to the recommended settings of the original RAD algorithm and further verified through parameter searching in the proposed AO system. The initial value of $\zeta_{k-1}$ was set to 10^−16^, and in the k-th iteration it was defined as

(7) $$\zeta_{k-1}=Max(10^{-6},1-\beta_{2}^{k})$$

In summary, RAD-SPGD retains the advantages of SPGD, including independence from a wavefront sensor, simple implementation, and strong robustness. Meanwhile, by introducing adaptive gain regulation, bias correction, and a constraint on the parameter update velocity, the proposed method can effectively reduce the influence of abnormal gradient perturbations and improve the stability and convergence speed of closed-loop correction in wavefront sensorless adaptive optics systems.

## 3. System Description

**Figure 1:**
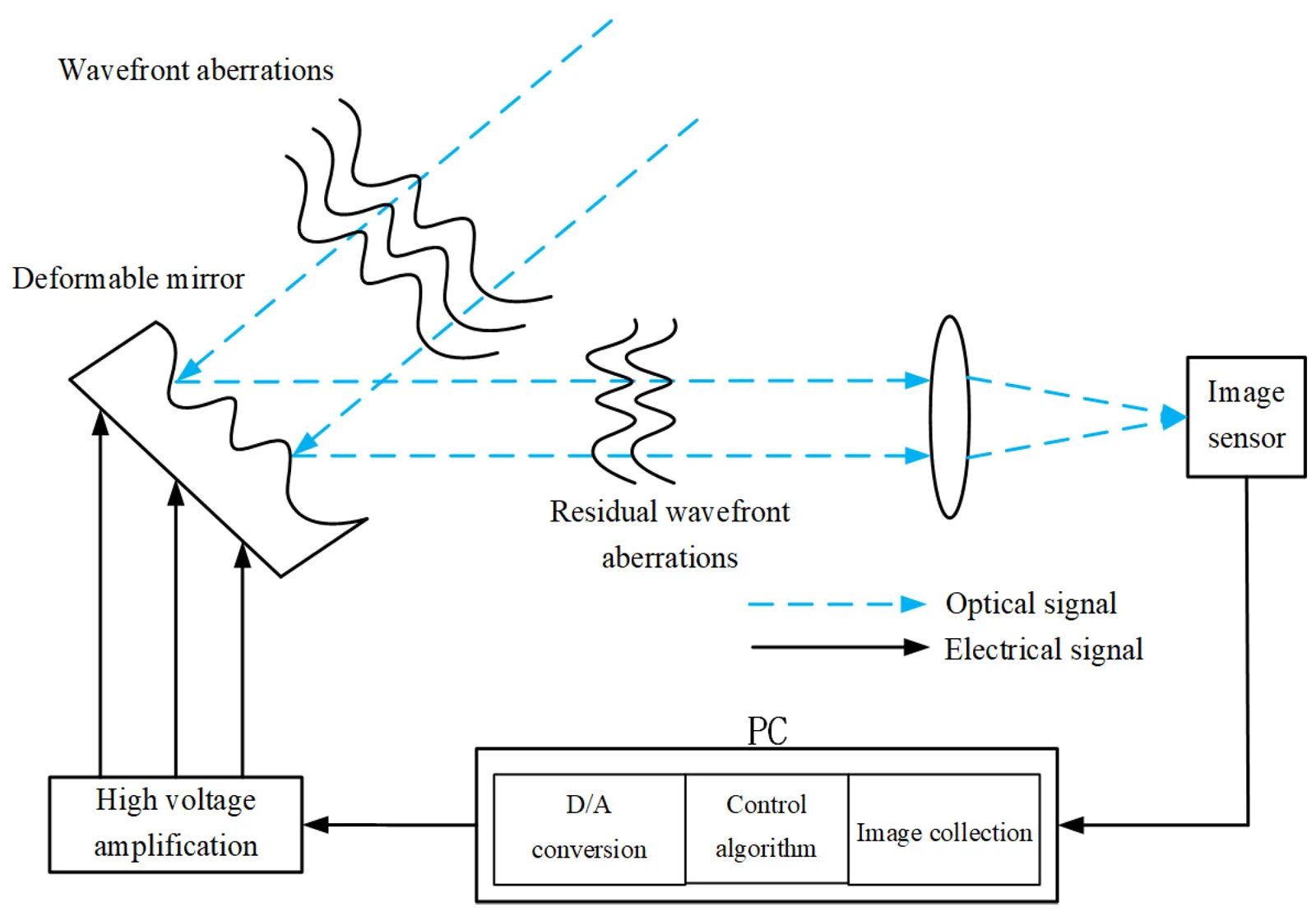
The wavefront sensorless AO system.

The RAD-SPGD-based wavefront sensorless AO system is shown in Figure 1. It mainly consists of a wavefront corrector, namely a 97-element MEMS deformable mirror, an image sensor, and a control module. The distorted wavefront is reflected by the deformable mirror and then imaged onto the image sensor through an imaging lens. The control module acquires the imaging information from the image sensor and generates the control signals for the deformable mirror using the RAD-SPGD control method. The control signals are applied to the actuators of the deformable mirror through a high-voltage amplifier, thereby producing a compensating phase. Subsequently, the residual wavefront is used as the input for further closed-loop correction until the predefined condition is satisfied.

According to the operating principle of the deformable mirror, the compensating surface profile ϕ(p,q) generated by the deformable mirror can be expressed as a linear combination of the influence functions of all actuators:

(8) $$\phi (p,q)=\sum_{i=1}^{N}u_{i}S_{i}(p,q)$$

where $S_{i}(p,q)$ is the influence function of the (i)-th actuator, and $u_{i}$ is the control voltage applied to the (i)-th actuator.

In the simulations, the Strehl ratio (SR) and mean radius (MR) were used as performance metrics to evaluate the correction performance of the AO system [17,18]. The SR is defined as follows:

(9) $$SR=\frac{Max(I(m,n))}{Max(I_{0}(m,n))}$$

where I(m,n) is the far-field intensity distribution corresponding to the aberrated wavefront, and $I_{0}(m,n)$ is the far-field intensity distribution corresponding to the ideal plane wavefront. A larger SR indicates better correction capability of the AO system, and the maximum value of SR is 1.

The MR is defined as follows:

(10) $$MR=\frac{\iint \sqrt{{\left(m-m_{0}\right)}^{2}+{\left(n-n_{0}\right)}^{2}}I(m,n)dmdn}{\iint I(m,n)dmdn}$$

where $\sqrt{{\left(m-m_{0}\right)}^{2}+{\left(n-n_{0}\right)}^{2}}$ represents the distance between the spot coordinate (m,n) and the centroid $\left(m_{0},n_{0}\right)$ on the image plane. A smaller MR indicates a smaller aberration.

In this study, SR was adopted as the performance metric for the static aberration correction simulations, whereas MR was used for the dynamic aberration correction simulations and experimental validation to assess the correction performance of RAD-SPGD.

## 4. Simulation Results and Analysis

### 4.1. Static Aberration Correction Simulation

The wavefront aberrations to be corrected were generated using Roddier’s method to produce multiple phase screens [19]. These phase screens followed the Kolmogorov power spectral model, were mutually uncorrelated, and consisted of Zernike modes from the 3rd to the 104th order, excluding the tip and tilt terms. The turbulence strength of the distorted wavefront was characterized by $D/r_{0}$, where D denotes the telescope aperture diameter and $r_{0}$ represents the atmospheric coherence length. A 97-element MEMS deformable mirror was employed as the wavefront corrector to evaluate the correction capability of the RAD-SPGD control method under different turbulence conditions.

**Figure 2:**
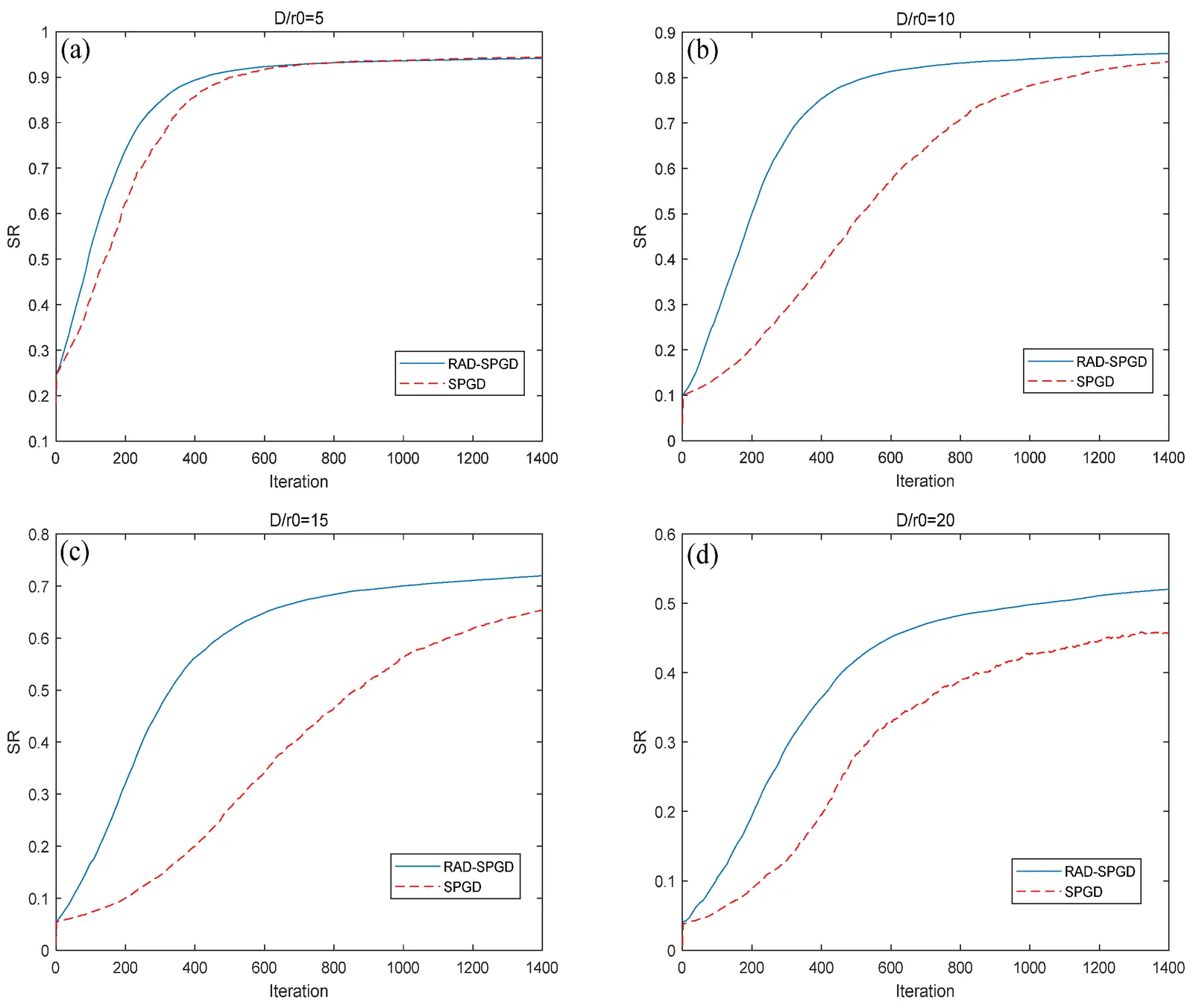
Comparison of the SR convergence curves for different algorithms under turbulence strengths of (**a**) 5, (**b**) 10, (**c**) 15, and (**d**) 20.

In the simulation experiments, 100 independent frames of randomly generated wavefront aberrations were selected as correction targets under four turbulence levels, corresponding to $D/r_{0}$ = 5, 10, 15 and 20. The average correction performance over these 100 independent aberration realizations was used as the evaluation criterion. To ensure a fair comparison, the optimal parameters of each algorithm under different turbulence conditions were determined through extensive preliminary experiments. The maximum number of iterations was uniformly set to 1400 for all algorithms. All simulation results presented below were obtained by averaging the correction performance over 100 independent wavefront aberration realizations under each turbulence condition. The algorithms were then compared in terms of convergence speed and correction accuracy. The corresponding convergence curves are shown in Figure 2.

As shown in Figure 2, RAD-SPGD achieved faster convergence under all turbulence conditions. The convergence speed was evaluated based on the number of iterations required to reach 80% of the maximum SR. Compared with conventional SPGD, RAD-SPGD consistently reached this criterion with fewer iterations, demonstrating improved optimization efficiency. Although SPGD can eventually reach a similar final SR level under some turbulence conditions, it requires more iterations to achieve the same correction level compared with RAD-SPGD. The maximum SR value was determined from the stable convergence stage under each turbulence condition. The convergence speed improvement was calculated by comparing the reduction in convergence iterations required by RAD-SPGD and SPGD to reach the same convergence criterion.

Under weak turbulence, as shown in Figure 2a, RAD-SPGD reduced the number of iterations by approximately 47% compared with SPGD. When $D/r_{0}=10$, as shown in Figure 2b, this advantage became more pronounced, with the required number of iterations reduced by approximately 60%. For $D/r_{0}=15$, as shown in Figure 2c, RAD-SPGD maintained a significant advantage, reducing the number of iterations by approximately 60% relative to SPGD. Even under the strong turbulence condition of $D/r_{0}=20$, as shown in Figure 2d, where the convergence accuracy and speed of all algorithms decreased due to the limited correction capability of the deformable mirror, RAD-SPGD still outperforms the other methods throughout the entire iterative process, reducing the number of iterations by approximately 58% compared with SPGD.

**Figure 3:**
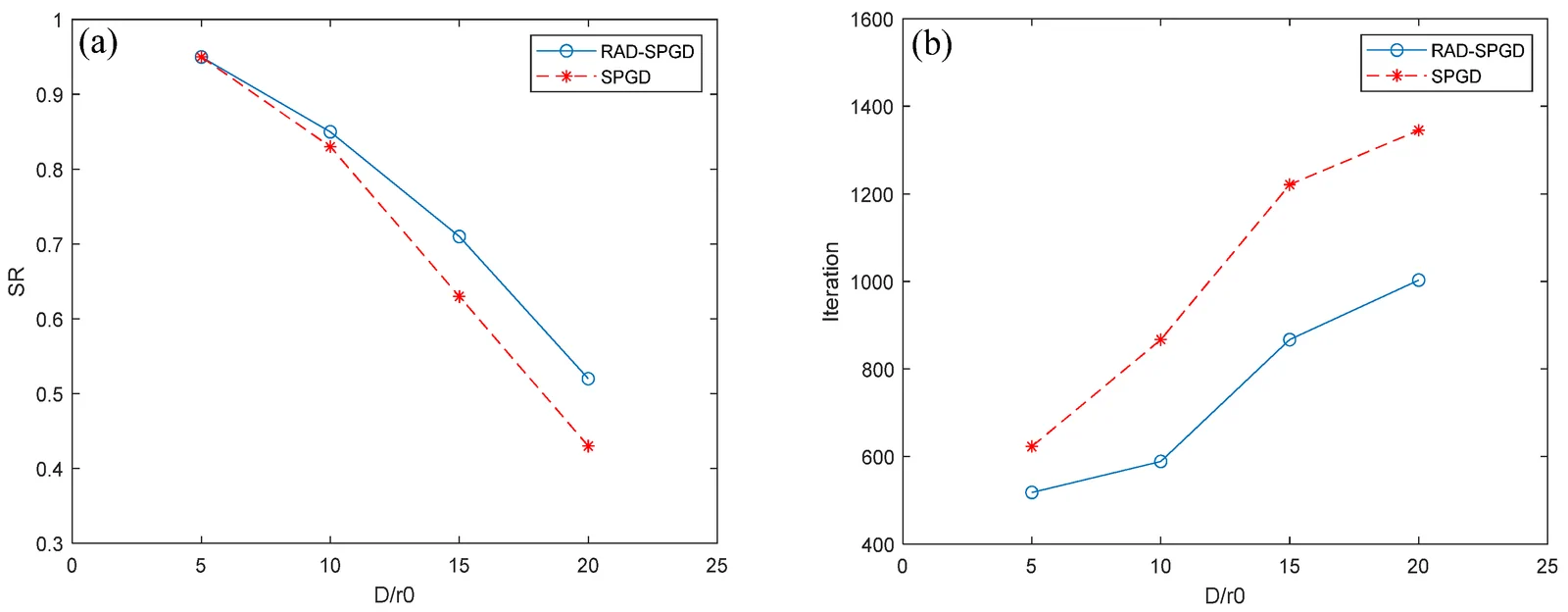
Comparison of SR and iterations under different turbulence conditions: (**a**) SR comparison and (**b**) iteration comparison.

After 1400 iterations, the convergence results of the SR values obtained by the two algorithms under different turbulence conditions are shown in Figure 3a. As shown in Figure 3a, when the turbulence level is relatively weak, the convergence values of the two methods are close to each other. However, as the turbulence strength increases, the correction capability of the RAD-SPGD-based AO system becomes significantly better than that of the SPGD-based AO system under the same number of iterations.

Figure 3b compares the number of iterations required to reach 80% of the maximum SR. The results show that, with increasing turbulence strength, the iteration requirement of the SPGD-based AO system increases markedly to achieve the same correction capability, while the RAD-SPGD-based AO system maintains a clear advantage in convergence speed.

### 4.2. Dynamic Aberration Correction Simulation

In the dynamic aberration correction simulation, dynamic wavefront aberrations collected from a near-sea-surface experimental site were used as the correction objects to evaluate the correction performance of different control algorithms. A laser with a wavelength of 808 nm was horizontally transmitted over a distance of 1 km and then focused onto the detection plane by an imaging system with an equivalent focal length of 5 m. Under this configuration, wavefront sequences under different turbulence conditions were continuously acquired using a Hartmann wavefront sensor. Based on the Hartmann wavefront sensor, dynamic wavefront sequences under different turbulence conditions were acquired at a sampling rate of approximately 167 Hz. A total of 10,000 wavefront frames were recorded, and a continuous segment of the acquired sequence was selected for evaluating the correction performance of different algorithms.

Considering that tip and tilt aberrations are usually corrected by an independent correction loop in practical applications, the tip and tilt components were removed from the collected dynamic wavefront distortions. Two groups of distorted wavefronts with average root mean square (RMS) wavefront errors of 0.13 λ and 0.38 λ were selected as the correction objects, where λ denotes the wavelength. These data can effectively represent the characteristics of distorted wavefronts affected by atmospheric turbulence in practical scenarios, thereby providing a reliable basis for verifying the adaptability of different algorithms under complex conditions.

**Figure 4:**
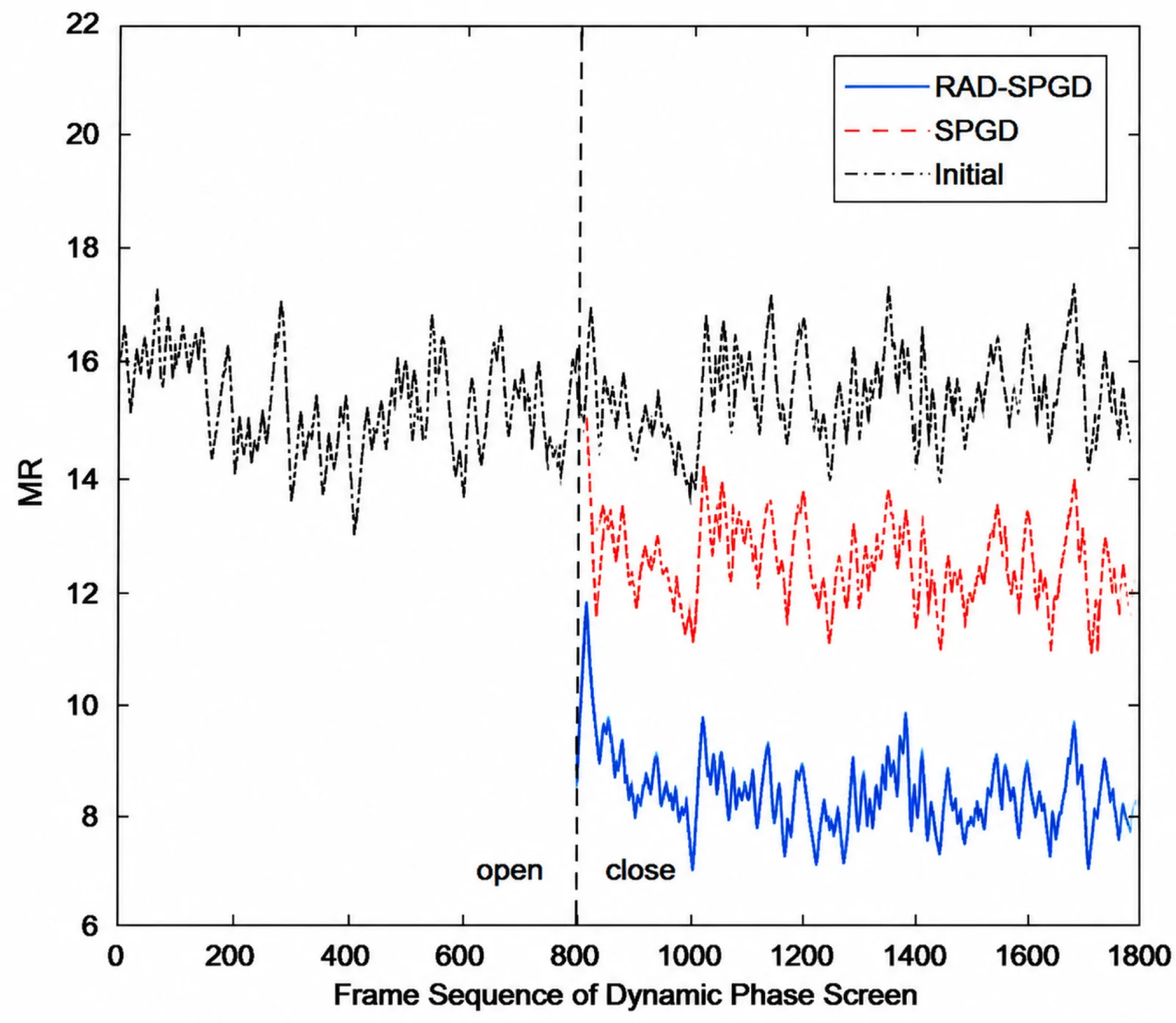
Comparison of MR during correction when the average RMS wavefront error is 0.13 λ.

**Figure 5:**
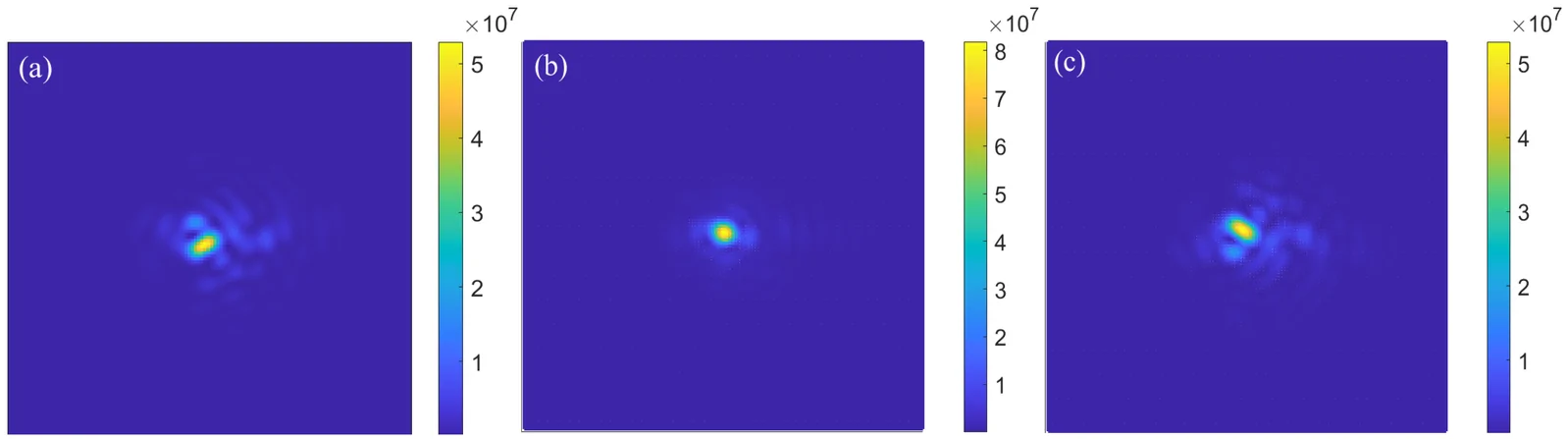
Intensity distributions before and after correction with an RMS wavefront error of 0.13 λ: (**a**) before correction, (**b**) RAD-SPGD, and (**c**) SPGD.

Figure 4 and Figure 5 show the MR variation curves during the correction process and the corresponding multi-frame averaged focal spot images before and after correction for different methods when the average RMS wavefront error is 0.13 λ. As shown in Figure 4, under the open-loop condition, represented by the dash-dotted line, the MR value of the focal spot remained at a relatively high level and exhibited large fluctuations. Before the activation of the correction algorithms, the system operated under an open-loop condition, where the deformable mirror maintained its initial state without receiving feedback control signals. At the 800th frame, the RAD-SPGD and SPGD algorithms were introduced for closed-loop correction. By comparing the MR values from the 1400th to the 1800th frame, it can be observed that the RAD-SPGD algorithm, represented by the solid line, outperforms SPGD, represented by the dashed line, in terms of both convergence speed and stability. After the closed-loop correction was introduced, the MR value of RAD-SPGD rapidly stabilized at approximately 8, whereas that of SPGD stabilized at approximately 12, indicating that RAD-SPGD achieved a significant improvement in correction performance compared with SPGD.

This improvement is mainly attributed to the increased dependence of the algorithm on single-iteration gradient estimation under rapidly varying atmospheric turbulence conditions. Under dynamic turbulence conditions, the conventional SPGD algorithm is easily affected by perturbations, and its fixed gain coefficient has limited adaptability to turbulence variations, resulting in slow convergence and local oscillations in the correction curve. In contrast, RAD-SPGD introduces a conformal symplectic integrator, which effectively suppresses oscillations. Meanwhile, its adaptive step-size adjustment strategy further improves the convergence speed. In addition, Figure 5 shows that both algorithms can reduce the dispersion of the focal spot and make the spot more concentrated. Among them, the focal spot corrected by RAD-SPGD exhibits the best performance, with the smallest degree of spot dispersion.

**Figure 6:**
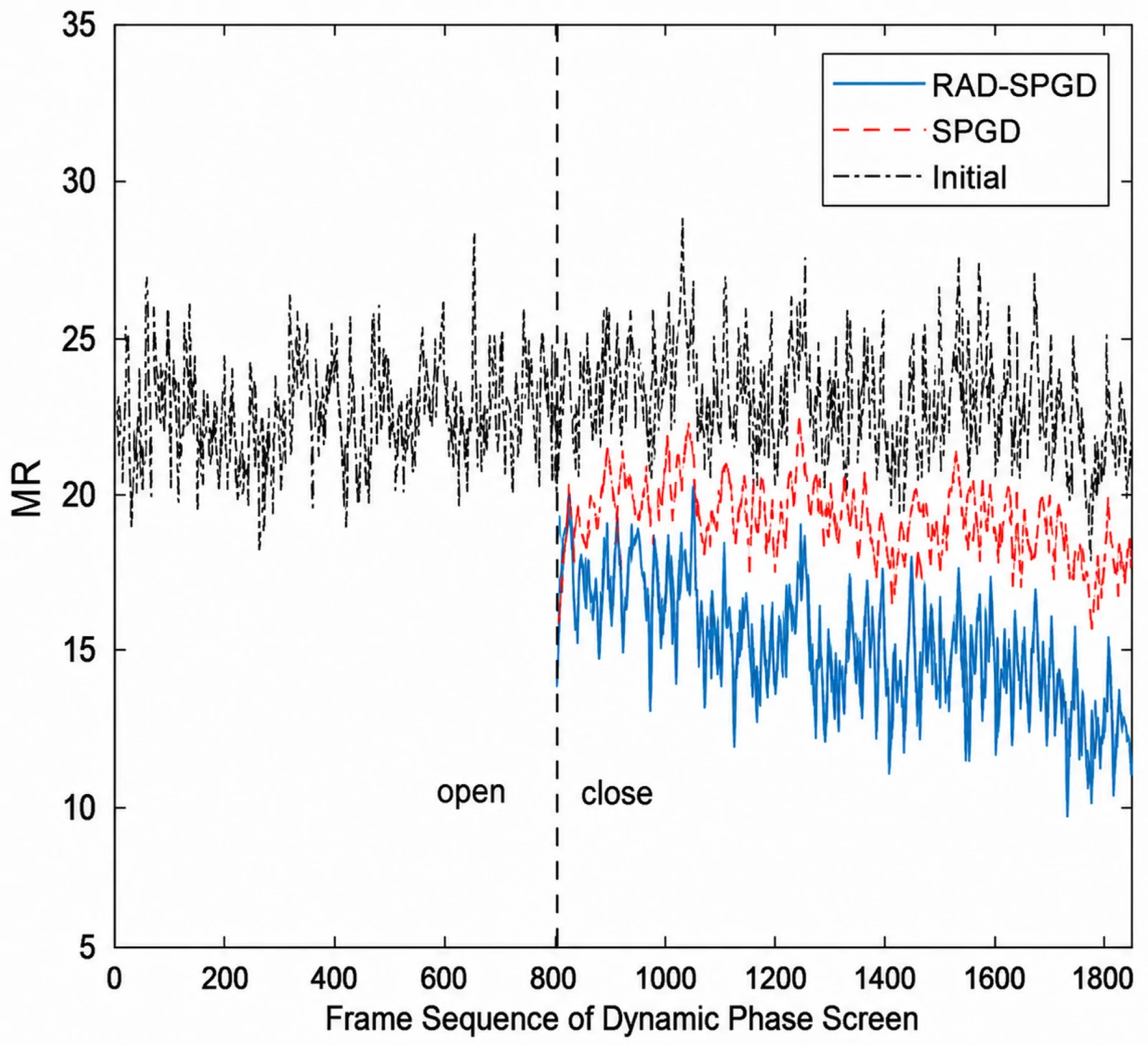
Comparison of MR during correction when the average RMS wavefront error is 0.38 λ.

**Figure 7:**
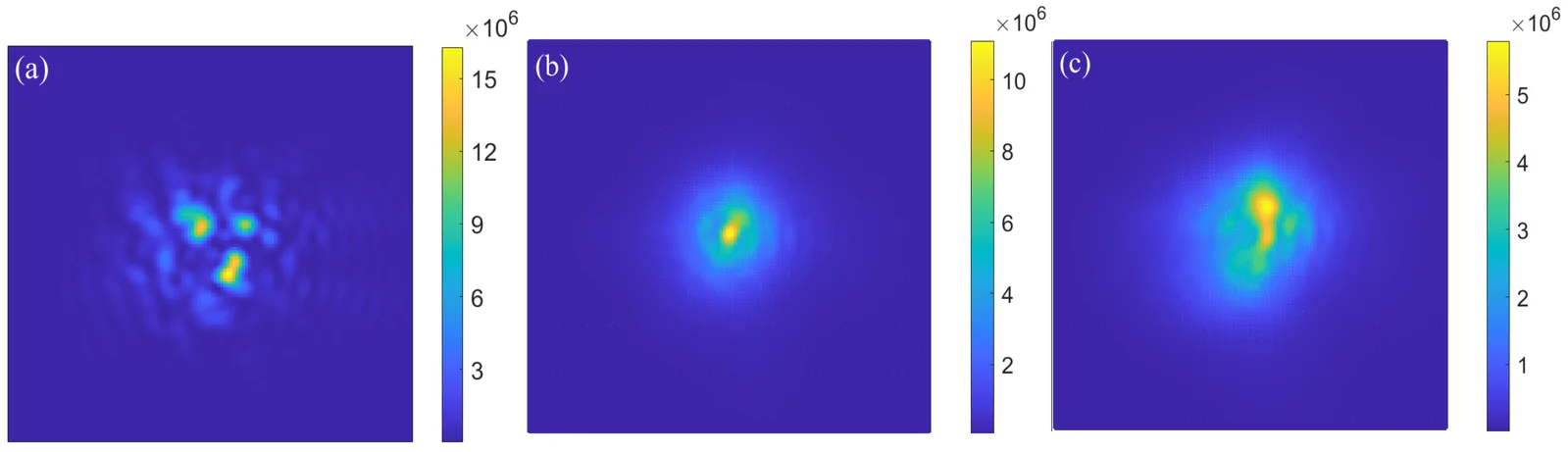
Intensity distributions before and after correction with an RMS wavefront error of 0.38 λ: (**a**) before correction, (**b**) RAD-SPGD, and (**c**) SPGD.

Figure 6 and Figure 7 show the MR variation curves during the correction process and the corresponding multi-frame averaged focal spot images before and after correction for different methods when the average RMS wavefront error is 0.38 λ.

As shown in Figure 6, before correction, the MR value fluctuated around 23. After closed-loop correction was introduced, RAD-SPGD reduced the MR value to approximately 13, whereas SPGD reduced it to approximately 17, indicating that RAD-SPGD improved the correction performance by approximately 23.5% compared with SPGD. The comparison of the focal spot images before and after correction in Figure 7 shows that both methods can effectively reduce spot dispersion when the aberration increases. However, the focal spot corrected by RAD-SPGD is more concentrated and exhibits a smaller degree of dispersion.

## 5. Experimental Results and Analysis

Physical experiments were conducted under the quasi-static system aberration inherent to the experimental platform to validate the proposed algorithm. In the physical experiment, the quasi-static system aberration is mainly composed of the initial surface deformation of the deformable mirror, residual optical aberrations, and alignment errors of the experimental platform. A 97-element MEMS deformable mirror (DM97-25, ALPAO) was employed as the wavefront corrector, and its main specifications are listed in Table 1. The deformable mirror consists of 97 actuators that generate continuous mirror surface deformation for wavefront aberration compensation.

**Figure 8:**
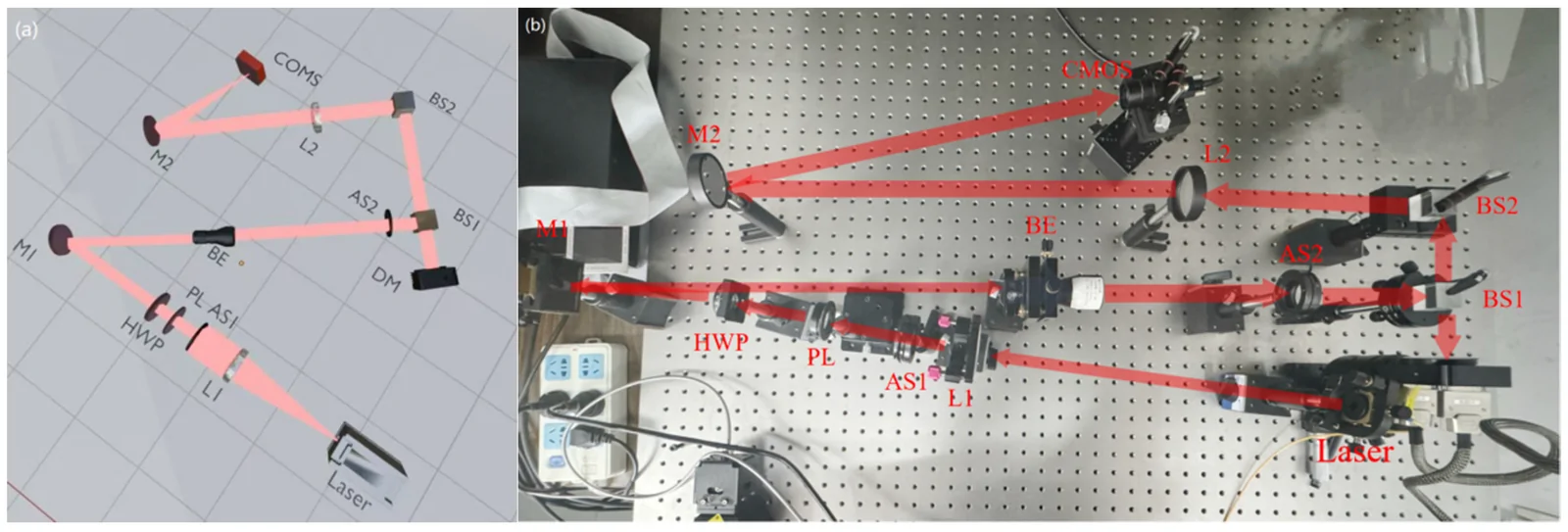
Experimental optical system: (**a**) optical schematic and (**b**) experimental setup.

The experimental optical system is shown in Figure 8, where Figure 8a illustrates the optical layout and Figure 8b presents the experimental setup. The laser beam passes through lens L1, aperture stop AS1, polarizer PL, and half-wave plate (HWP), and is then reflected by mirror M1. After passing through a 2.5× beam expander (BE), the expanded beam is reflected by beam splitter BS1 onto the deformable mirror (DM). The reflected beam subsequently passes through beam splitter BS2 and is focused by lens L2 (f = 1000 mm), before being reflected by mirror M2 onto the camera sensor. The polarizer is used to suppress the polarization noise of the laser source, while the HWP ensures proper beam polarization before reflection by M1. Under this optical configuration, the diffraction-limited Airy spot corresponds to an MR value of approximately 28 pixels.

The spot image acquired by the camera is transmitted to a computer in real time. A Prime BSI Express camera was used for focal spot image acquisition in the experiment. The camera is equipped with a 4.2-megapixel sensor with a sensor size of 13.3 mm × 13.3 mm, and the maximum acquisition frame rate is 43 fps under 16-bit operation mode. During data processing, a region of interest (ROI) was selected to facilitate focal spot visualization and MR calculation. The control algorithm calculates the image quality metric (MR) and generates the corresponding driving signals for the deformable mirror. The control signals are output through a D/A conversion board, amplified by a high-voltage amplifier, and applied to the 97 actuators of the deformable mirror to produce the required mirror surface deformation. The algorithm iteratively updates the actuator voltages until the preset maximum number of iterations or the convergence criterion is reached. All control algorithms were implemented in MATLAB R2022b.

During the closed-loop correction experiments, the 97-element MEMS deformable mirror served as the wavefront correction element, and its correction performance was jointly determined by the responses of all actuators under the applied control voltages. Based on the focal-plane spot image acquired by the camera, the control algorithm calculated the image quality metric (MR) and iteratively updated the control voltages of all actuators with the objective of minimizing the MR value. As the control voltages were continuously adjusted, the deformable mirror generated a continuous mirror surface deformation to compensate for the aberrated wavefront, thereby reducing the residual wavefront error and gradually concentrating the focal-plane spot. Therefore, the converged MR value, convergence speed, spot concentration after correction, and peak intensity were adopted to evaluate the closed-loop correction performance of the 97-element MEMS deformable mirror under different control algorithms.

**Figure 9:**
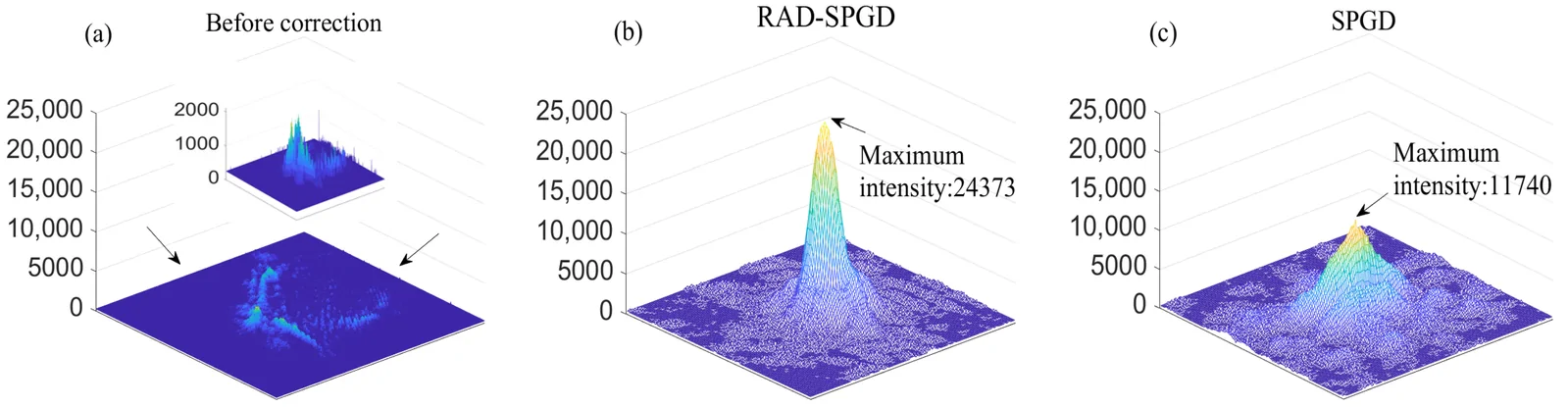
Comparison of intensity distributions before and after correction: (**a**) before correction, (**b**) RAD-SPGD, and (**c**) SPGD.

**Figure 10:**
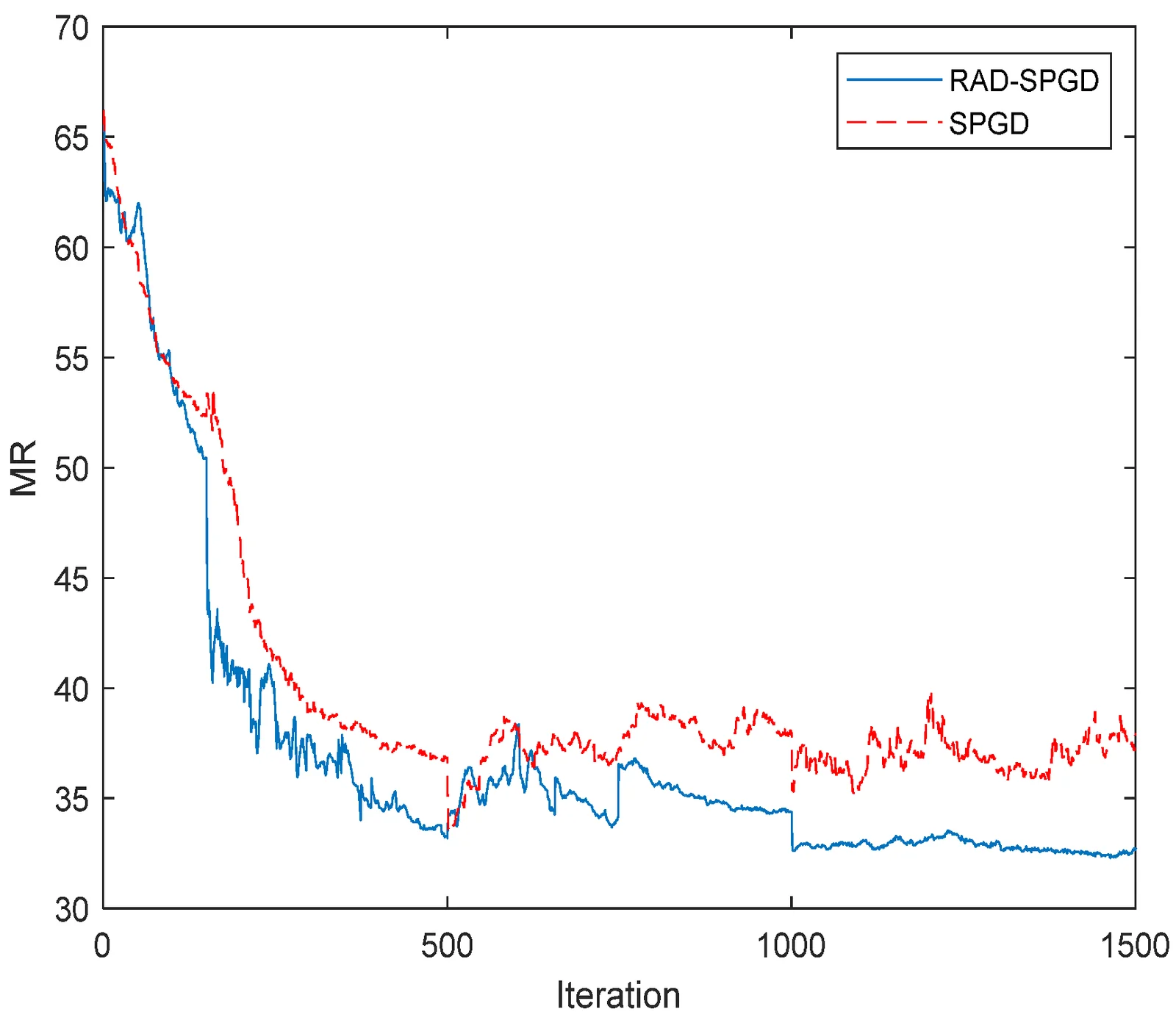
Comparison of MR curves during correction.

In the experiments, RAD-SPGD and SPGD were used as the control algorithms to correct the distorted wavefronts. The experimental results correspond to a single independent trial under fixed optical and acquisition conditions, and the same conditions were maintained for both RAD-SPGD and SPGD to ensure a fair comparison. The focal spot images before and after correction and the MR convergence curves are shown in Figure 9 and Figure 10, respectively. As shown in Figure 9 and Figure 10, RAD-SPGD outperforms SPGD in terms of both the focal spot distribution and the convergence behavior.

In Figure 9, the maximum pixel intensity value of the corrected focal spot image obtained by the RAD-SPGD-based wavefront sensorless AO system was 24,373, which was higher than that obtained by SPGD (11,740). In addition, the focal spot corrected by RAD-SPGD was more concentrated and exhibited a smaller degree of dispersion. In this study, peak intensity is defined as the maximum value of the focal plane captured by the camera. All images acquired via the RAD-SPGD and SPGD algorithms were obtained under identical experimental conditions, including consistent optical configuration, camera parameters and exposure settings. No intensity normalization was performed on the collected raw images. As shown in Figure 10, both algorithms achieved convergence after 1500 iterations. After correction, the MR value of RAD-SPGD decreased from 66.0644 to 32.74, whereas that of SPGD decreased to 37.12. Moreover, RAD-SPGD approached convergence after approximately 1000 iterations, while SPGD required approximately 1400 iterations to converge. Therefore, the correction speed of RAD-SPGD was improved by approximately 28.6% compared with SPGD. Overall, RAD-SPGD demonstrated obvious advantages over SPGD in terms of both correction performance and convergence speed.

To further evaluate the practical implementation capability of the proposed algorithm, the real-time performance and closed-loop bandwidth of the experimental system were further analyzed.

During the implementation of the algorithm, one iteration mainly consists of perturbation signal loading, deformable mirror response, camera acquisition, performance metrics, and control update. The software environment is MATLAB R2022b. Among these factors, the camera acquisition frame rate and the interpreted execution process of MATLAB (version R2022b) play dominant roles in determining the convergence speed of the system. When the hardware and software configurations are fixed, the convergence performance of different control algorithms can be compared under the same experimental conditions.

For the current experimental system, the camera frame rate is 43 fps, and the response time of the deformable mirror is 1.5 ms. The effective closed-loop correction bandwidth of the system was estimated using the following equation:

(11) $$f_{s}\leq \frac{f_{c}}{30\sqrt{N}}$$

where the camera frame rate is $f_{c}$ = 43 fps and the number of deformable mirror actuators is *N* = 97. The calculated theoretical effective correction bandwidth of the system is approximately 0.15 Hz.

The current experimental platform is mainly used to verify the correction performance and convergence advantages of the RAD-SPGD algorithm, rather than to achieve high-speed real-time control. In future work, high-speed cameras, FPGA, or GPU-based hardware acceleration schemes will be considered to optimize image processing and control computation, reduce the iteration time, and further improve the closed-loop bandwidth to achieve faster wavefront correction.

## 6. Conclusions

A relativistic adaptive gradient descent-based stochastic parallel gradient descent (RAD-SPGD) algorithm was proposed for the closed-loop control of a 97-element MEMS deformable mirror in a wavefront sensorless adaptive optics system. Numerical simulations and physical experiments were conducted to evaluate its convergence performance and correction capability under different turbulence conditions.

Compared with the conventional SPGD algorithm, the proposed RAD-SPGD algorithm achieved faster convergence while maintaining comparable or superior correction performance. In particular, the correction speed was improved by approximately 47%, 62%, 60%, and 58% under turbulence strengths of 5, 10, 15, and 20, respectively. In addition, RAD-SPGD exhibited better correction stability and adaptability in dynamic aberration correction. Physical experiments further verified its effectiveness, with the MR converging to 32.74, which is closer to the diffraction-limited value than that achieved by the conventional SPGD algorithm. These results demonstrate that RAD-SPGD effectively enhances the closed-loop control performance of multi-actuator MEMS deformable mirrors and provides an efficient approach for a real-time wavefront sensorless adaptive optics system. In this work, the conventional SPGD algorithm was selected as the baseline method because it is a widely used control algorithm in wavefront sensorless adaptive optics systems. The main objective of this study is to investigate the improvement introduced by integrating the RAD optimizer into the SPGD framework. Future work will further investigate the systematic comparison between RAD-SPGD and other adaptive control methods.

## Figures and Tables

**Table 1:** Specifications of the DM97-25 MEMS deformable mirror.

Model	Drives Number	Drives Spacing (mm)	Aperture Diameter (mm)	Radial Drives Number	Defocusing /Astigmatic (um)	Stable Time (ms)	Size (mm)
DM97-25	97	2.5	22.5	11	30	1.5	62 × 84 × 23

## Data Availability

The data presented in this study are available from the corresponding author upon reasonable request. The data are not publicly available because they are part of an ongoing research project.
